# Isolation and biogeography of the oligotrophic ocean diazotroph, *Crocosphaera waterburyi* nov. sp

**DOI:** 10.1093/ismejo/wrae217

**Published:** 2024-10-23

**Authors:** Catie S Cleveland, Kendra A Turk-Kubo, Yiming Zhao, Jonathan P Zehr, Eric A Webb

**Affiliations:** Marine and Environmental Biology, University of Southern California, 3616 Trousdale Pkwy, Los Angeles, CA, 90089, United States; Ocean Sciences Department, University of California, Santa Cruz, 1156 High St., Santa Cruz, CA, 95064, United States; Marine and Environmental Biology, University of Southern California, 3616 Trousdale Pkwy, Los Angeles, CA, 90089, United States; Ocean Sciences Department, University of California, Santa Cruz, 1156 High St., Santa Cruz, CA, 95064, United States; Marine and Environmental Biology, University of Southern California, 3616 Trousdale Pkwy, Los Angeles, CA, 90089, United States

**Keywords:** nitrogen fixation, cyanobacteria, oligotrophic oceans, *Crocosphaera*

## Abstract

Marine N_2_-fixing cyanobacteria, including the unicellular genus *Crocosphaera*, are considered keystone species in marine food webs. *Crocosphaera* are globally distributed and provide new sources of nitrogen and carbon, which fuel oligotrophic microbial communities and upper trophic levels. Despite their ecosystem importance, only one pelagic, oligotrophic, phycoerythrin-rich species, *Crocosphaera watsonii*, has ever been identified and characterized as widespread. Herein, we present a new species, named *Crocosphaera waterburyi*, enriched from the North Pacific Ocean. *C. waterburyi* was found to be phenotypically and genotypically distinct from *C. watsonii*, active *in situ*, distributed globally, and preferred warmer temperatures in culture and the ocean. Additionally, *C. waterburyi* was detectable in 150- and 4000-meter sediment export traps, had a relatively larger biovolume than *C. watsonii*, and appeared to aggregate in the environment and laboratory culture. Therefore, it represents an additional, previously unknown link between atmospheric CO_2_ and N_2_ gas and deep ocean carbon and nitrogen export and sequestration.

## Introduction

N_2_-fixing cyanobacteria are widespread members of the global oceans and are impactful on the overall health and function of marine ecosystems [1, 2]. Members of the unicellular cyanobacterial genus *Crocosphaera* are photosynthetic, phycocyanin, or phycoerythrin-rich bacteria that convert N_2_ gas from the atmosphere into bioavailable forms using the enzyme nitrogenase (encoded by the genes *nifH*, *nifD*, and *nifK*) [2–4]*.* Currently, *Crocosphaera* have been described from various biogeographical regions including coastal waters and the oligotrophic oceans [4–6]. The colors of various *Crocosphaera* are indicative of their ecological niches, with the phycocyanin-rich species harvesting red light common in benthic coastal habitats and phycoerythrin-rich strains harvesting blue light available in oligotrophic ocean waters [7]. The coastal, phycocyanin-rich *Crocosphaera* species include: *Crocosphaera subtropica*, *Crocosphaera chwakensis*, and *Cyanothece* sp. BG0011. Prior to this study, the phycoerythrin-rich *Crocosphaera* included only one valid species, *Crocosphaera watsonii*, which was the only known abundant, unicellular, free-living, N_2_-fixing cyanobacterium in the oligotrophic oceans [2, 5, 6].

*C. watsonii* generates bioavailable nitrogen (N) and carbon (C) and impacts biogeochemical cycling in broad regions [2, 4, 6]. New C from *Crocosphaera* can provide a resource for upper trophic levels and allows for microbial recycling processes to take place, whereas new N fuels N-limited phytoplankton that drive the biological C pump [2, 8]. During summer in the upper euphotic zone of the North Pacific Subtropical Gyre, *C. watsonii nifH* gene-based abundances can be found at higher copy number than other diazotrophs at 9.4 ± 0.7 × 10^5^ to 2.8 ± 0.9 × 10^6^  *nifH* copies per L [9]. Recent work has also shown that *Crocosphaera* can also have both direct and indirect impacts on N + C export to the deep ocean [10–14]. Deep C export is a mitigating factor in the ocean response to rising anthropogenic CO_2_ conditions. Thus, defining the role that *Crocosphaera* plays in both production and export will improve understanding of how the oligotrophic oceans will be impacted by climate change.

In this study, we present the discovery and characterization of an oligotrophic species within genus *Crocosphaera*, named *Crocosphaera waterburyi* Cleveland and Webb nov. sp., (henceforth, *C. waterburyi*). The *C. waterburyi* Alani8 enrichment was obtained from oligotrophic waters in the North Pacific Ocean near Hawaii. Environmental *nifH* and metagenomic datasets showed that *C. waterburyi* was globally distributed in multiple oceans, contributed to C + N export, could be present and active deeper in the water column, exhibited a warm temperature optimum, and had a relatively large biovolume. *C. waterburyi* cells were also rod-shaped (vs spherical *C. watsonii*), ~5 μm in length by ~2 μm wide, phycoerythrin-rich, and formed large cellular aggregates. The assembled genome of *C. waterburyi* was comparable in size and GC content with *C. watsonii* strains, yet clustered in a distinct clade when compared by multiple metrics. Our characterization of *C. waterburyi* shows it as a previously overlooked, ecologically relevant taxa in oligotrophic ocean regions.

## Materials and methods

### Isolation and cultivation

A single isolate of *C. waterburyi,* strain Alani8, was enriched during the 2010 10-day R/V Kilo Moana KM-1013 cruise near Station ALOHA (22° 45′N, 158° 00′W) [15, 16]. The enrichment was started from a single, hand-picked *Trichodesmium* colony and incubated in YBCII media without vitamins [17] at 26°C in a Percival Incubator (Percival Scientific Inc., Perry, IA, United States; 12:12 Light:Dark cycle at ~100 μmol Q m^−2^ s^−1^). After ~30 days, the *Trichodesmium* colony had lysed, and the culture began to turn orange, suggesting the presence of a phycoerythrin-rich cyanobacterium. Samples from these enrichments were concentrated, streaked on parafilm-sealed 1.5% Type VII agarose plates (Sigma-Aldrich, Burlington, MA,), and incubated as above for >30 days. This process was repeated twice, and single colonies were picked to obtain unialgal enrichments. Cultures were non-axenic and were maintained in maximum log growth via weekly transfers to keep heterotrophs in low abundance based on previous *Crocosphaera* culturing work [5]. Cultures are available to order by the name “*Crocosphaera waterburyi*” under accession number “CCMP 3753” from the Provasoli-Guillard National Center for Marine Algae and Microbiota (NCMA) at Bigelow laboratories (https://ncma.bigelow.org/).

Wet mount epi-fluorescent and bright field microscopy with Zeiss DAPI and Cy3 filters, a Zeiss AxioStar microscope, and a Zeiss HBO50 light source (Zeiss, Oberkochen, Germany) were used to describe the cellular morphology, cellular biovolume, and pigmentation. Biovolume was determined using cell size measurements on ImageJ [18] and pigmentation was further analyzed with chlorophyll extractions (Supplemental Methods), [19]. Scanning electron microscopic (SEM) images were also taken to provide higher resolution of cellular morphology (Supplemental Methods).

### Extraction and sequencing

To concentrate biomass for DNA extraction, 100 ml of mid-log culture was centrifuged at 13000 RPM for 2 min at 25°C to form a pellet. DNA was then extracted using the Qiagen DNeasy PowerBiofilm kit (Qiagen, Germantown, MD, United States) following the manufacturer’s protocol with the following modifications: after addition of the cell material to the bead beating tube, the cells were lysed with liquid N_2_ freeze-thaws (5X), tube agitation (3X), and 65°C overnight Proteinase K (~1 ng/μl final concentration in 350 μl of Qiagen buffers MBL and 100 μl of FB; VWR International, Radnor, PA, United States) incubation. DNA was quantified using a Qubit 4 fluorometer (ThermoScientific, Waltham, MA, United States), and 260/280 quality was verified with a NanoDrop 1000 spectrophotometer (Thermo Fisher Scientific, Waltham, MA, United States). Library preparation with the NEBNext DNA Library Prep Kit and PE150 sequencing at a depth of 1Gbp was completed at Novogene Inc. (Sacramento, CA, United States).

### Genome assembly

The reads were assembled on the open-source web page KBase (KBase.com) following the public narrative, “Genome Extraction for Shotgun Metagenomic Sequence Data” (https://narrative.kbase.us/narrative/24019), (see: Supplemental Methods for full pipeline).

### Phylogenetic tree construction

To place the *C. waterburyi* genome in context with other near relative genomes available in GenBank, accessions in order *Chroococcales* (including families *Aphanothecaceae* and *Microcystaceae* [20]) and genus *Cyanothece* were obtained from the NCBI assembly site. A phylogenomic tree with 350 genomes/MAGs was created using the GToTree v1.6.31 workflow and associated programs [21–26] with *Gloeobacter violaceus* PCC 7421 (GCA_000011385.1) as the root. Subsequently, another maximum likelihood tree was created using 35 representative assemblies closely related to *C. waterburyi*. The tree used 251 conserved cyanobacterial HMMs [25] with at least 50% of the HMMs required in each genome to be included in the tree. The output tree data from GToTree was piped into IQTree2 using the best model finder method and 1000 bootstraps to generate the final consensus tree [27, 28].

We additionally used NCBI-blastn to place the *C. waterburyi nifH* gene in an environmental context and to create a 16S rRNA gene tree of representative *Crocosphaera* isolates. The phylogenetic tree was created using the *nifH* gene sequences from *Crocosphaera* enrichment cultures and 250 *nifH* gene sequences identified by blastn as having high identity to the *C. waterburyi nifH* gene. For the 16S rRNA gene tree, the *C. waterburyi* 16S rRNA gene was assembled from the trimmed reads using Phyloflash [29] and compared to 16S rRNA genes sequenced from *Crocosphaera* cultures. The phylogenetic tree pipeline was as follows: combined sequences for each respective tree were aligned in Geneious [30] using Clustal Omega 1.2.2 [31], trimmed manually, and subsequent *nifH* and 16S rRNA gene trees were created using RAxML 8.2.11 [32] with a GTR GAMMA nucleotide model, rapid bootstrapping (1000 bootstraps), and the maximum likelihood tree algorithm. A world map with the collection coordinates of *nifH* amplicon sequences most closely related to *C. waterburyi* Alani8 was also visualized using R packages ggplot2 and tidyverse [33, 34].

### Pangenome analysis

We used the pan genomic pipeline in Anvi’o v7.1 [35, 36] to define the core and accessory genes of 10 *Crocosphaera* assemblies, including six *C. watsonii* strains (WH0003 (GCA_000235665.2), WH0005 (GCA_001050835.1), WH0402 (GCA_001039635.1), WH8501 (GCA_000167195.1), WH8502 (GCA_001039555.1), WH0401 (GCA_001039615.1)), *C. chwakensis* CCY0110 (GCA_000169335.1), *C. subtropica* ATCC 51142 (GCA_000017845.1), *Cyanothece* sp. BG0011 (GCA_003013815.1), and *C. waterburyi*. Two environmental MAGs, *Crocosphaera* sp. DT_26 (GCA_013215395.1) and *Crocosphaera* sp. ALOHA_ZT_9 (GCA_022448125.1), were excluded from the pangenome as they were not from isolated cultures [9, 11, 12, 14, 37, 38] and their physiology has not yet been characterized. All assemblies, beside *C. waterburyi*, were obtained from NCBI. Briefly, the genomes were reformatted and annotated with NCBI-COG20, Pfams v35, KEGG-KOfams v2020-12-23, and HMMER v3 [25, 39–41] to define the conserved gene content in each assembly. The pangenome was constructed using an MCL 2 threshold suitable for less-similar genomes [42], and the FastANI v1.32 [43] heatmap used an ANI lower threshold of 80% similarity. Genomes were ordered by ANI similarity, and gene clusters were aligned and ordered in Anvi’o v7.1 by presence or absence in the genomes.

### Temperature profile

*C. waterburyi* was grown in Percival incubators at temperatures between 20–38° under the following conditions: identical 3000 K warm white lights at 96 μmol Q m^−2^ s^−1^, 12:12 diel cycle in YBC II media without vitamins [17]. The growth rates of *C. waterburyi* Alani8 across 20–38°C and the growth rates of two representative large and small cell *C. watsonii* strains from a previous study [5] were compared by normalizing to percent maximal growth (0–100%) to account for differences in light level and culture medium. More details for these calculations are available in the Supplemental Methods, as well as additional methods for comparative growth rate and N_2_-fixation measurements from *C. watsonii* and *C. waterburyi* at 26°C.

### Environmental read-mapping

We used the *C. waterburyi*, *C. watsonii* WH0003, *C. chwakensis* CCY0110, *Cyanothece* sp. BG0011, and *C. subtropica* ATCC 51142 genomes as targets for read recruiting to 63 metagenome samples from 4000 m depth in the ALOHA Deep Trap Sequencing project (PRJNA482655; DeLong research group at University of Hawai’i and Simons Collaboration on Ocean Processes and Ecology), [11, 12, 14, 38, 44], Station ALOHA 150 m net trap metagenomes (PRJNA358725), [9, 37, 38], GO-SHIP surface metagenomes [45], and BioGEOTRACES metagenomes [46] to define the range of genus *Crocosphaera*.

Read recruitment was also done with 934 TaraOceans DNA samples [47–49] to the complete genomes for UCYN-A1 ALOHA (GCA_000025125.1) and UCYN-A2 CPSB-1 (GCA_020885515.1) and draft genomes for *C. waterburyi* Alani8 and *C. watsonii* WH0401 (GCA_001039615.1). The TaraOceans temperature metadata was also obtained from the European Nucleotide Archive (ENA).

Briefly, the pipeline for read recruitment was as follows: Bowtie2 v2.5.2 mapped reads to the contig set [50], Samtools v1.9 converted SAMs to BAMs [51], CoverM v0.6.1 filtered the BAMs at 98% identity (https://github.com/wwood/CoverM), and Anvi’o v7.1 visualized and parsed the results [36]. The mean coverage and % recruitment values were used as metrics of abundance, and % genomes detection was used for presence vs absence. For TaraOceans metagenomes, mean coverages were compared across surface samples where ≥1 genome was present at >1x mean coverage. More detailed interpretations of these different Anvi’o parameters are available at https://merenlab.org/2017/05/08/anvio-views/ as well as in previous studies [52, 53].

### Detection of *nifH* gene and transcripts in the North Pacific subtropical gyre

Samples for the determination of diazotroph community composition and activity were collected during the SCOPE-PARAGON I research expedition in the North Pacific Subtropical Gyre (NPSG) July 22–August 5, 2021 (R/V Kilo Moana). Three types of samples were collected: size fractionated seawater samples (DNA); diel seawater samples (RNA); and samples of particles sinking out of the euphotic zone (DNA/RNA). All seawater samples were collected from three depths, 25 meters, 150 meters, and the deep chlorophyll maximum (DCM: ~135 meters), using Niskin bottles mounted to a CTD rosette (SeaBird Scientific Bellevue, WA, United States), and transferred into acid-washed polycarbonate bottles or carboys. Large volume (20 L) seawater samples were filtered serially using gentle peristaltic pumping through the following filters: 100 μm nitex mesh (25 mm, MilliporeSigma, Burlington, MA, United States); 20 μm polycarbonate (25 mm, Sterlitech Corp., Auburn, WA, United States) 3.0 μm polyester (25 mm, Sterlitech Corp., Auburn, WA, United States); and 0.2 μm Supor (25 mm; Pall Corporation, Port Washington, NY, United States). Diel samples (2.5–4 L) were collected every ~6 hr over 30 h and filtered serially through 3.0 μm polyester (25 mm, Sterlitech Corp., Auburn, WA, United States) and 0.2 μm Supor filters (25 mm, Pall Corporation, Port Washington, NY, United States), with care taken to keep filtration times under 30 min.

Sinking particles were collected using surface tethered net traps (diameter 1.25 m, 50 μm mesh cod end), [54] and deployed at 150 m for 24 hr. Upon recovery of the net traps, particles were gently resuspended in sterile filtered 150 m water and split into multiple samples as previously described [55]. Particle slurries were gently filtered through 0.2-μm pore size Supor filters (25 mm; Pall Corporation). All filters were flash frozen in liquid N_2_ and stored at −80°C until extraction.

DNA and RNA were co-extracted from all samples using the AllPrep DNA/RNA Micro kit (Qiagen, Germantown, MD, United States) according to the manufacturers’ guidelines with modifications described previously [56]. RNA extracts were DNase digested using the Turbo DNA-free kit (Ambion, Austin, TX, United States) to remove any DNA contamination. Then, cDNA was synthesized with the Superscript IV First-Strand Synthesis System (Invitrogen, Waltham, MA, United States) and primed by universal *nifH* reverse primers nifH2, nifH3 using reaction conditions as previously described [57]. All DNA and RNA extracts were screened for purity using a NanoDrop spectrophotometer (ThermoScientific, Waltham, MA, United States), and DNA was quantified using Picogreen dsDNA Quantitation kit (Molecular Probes, Eugene, OR, United States).

Partial *nifH* fragments were PCR-amplified using the universal primers nifH1–4 [58, 59] and sequenced using high throughput amplicon sequencing as detailed previously [60]. Amplicon sequence variants (ASVs) were defined using the DADA2 pipeline [61] with customizations specific to the *nifH* gene (J. Magasin, https://github.com/jdmagasin/nifH_amplicons_DADA2). *Crocosphaera* ASVs were identified using blastx against a curated *nifH* genome database (https://www.jzehrlab.com/about-3), including ASVs 100% identical to *C. waterburyi* and *C. watsonii* WH8501 (AADV02000024.1).

## Results and discussion

### Morphological and physiological characteristics

**Figure 1:**
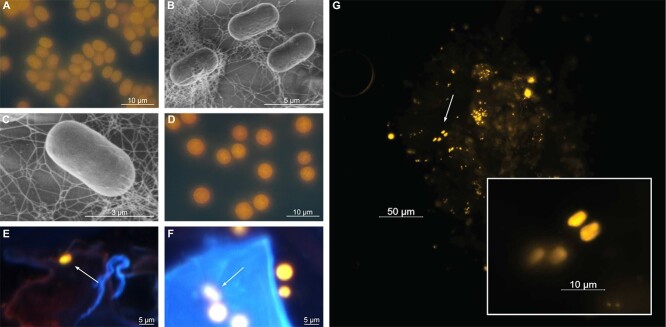
Pigmentation (**A, D**) as shown by DAPI-LP epifluorescence and morphology of *C. waterburyi* Alani8 by SEM (**B-C**). Environmental photos were taken using DAPI-LP excitation from 75 m depth net traps cells during the 2010 North Pacific RV Kilo Moana KM1013 cruise from which *C. waterburyi* was isolated (**E-F**). White arrows indicate *C. waterburyi*-like cells rod-shaped, phycoerythrin-rich cells. *C. waterburyi*-like cells, visualized by a Cy3 filter, are also shown attached to sinking particles caught in net traps during the 2021 SCOPE-PARAGON I research expedition (**G**).

Following isolation from the North Pacific near Station ALOHA, *C. waterburyi* consistently displayed cell morphology and pigmentation that bridged the gap between the coastal, phycocyanin-rich *C. subtropica*, *C. chwakensis*, and *Cyanothece* sp. BG0011 (CrocoG hereafter) with the oligotrophic, phycoerythrin-rich *C. watsonii*. Specifically, *C. waterburyi* was rod-shaped and ~5 μm long by ~2 μm wide like *Cyanothece* sp. BG0011 (Fig. 1A-C), [62]. However, although rod-shaped, they were still similar in cell size to larger cells of the spherical *C. watsonii* (~5 μm), (Fig. 1D) and were shown to be phycoerythrin-rich using DAPI-LP epifluorescence (Fig. 1A). *C. waterburyi* also formed aggregates in culture (i.e. flocs) embedded in exopolysaccharides like the coastal *Crocosphaera* species, and exhibited elongated rod shapes (Fig. 1A-C), [6]. *C. waterburyi*-like rod shaped, phycoerythrin-rich cells also appeared to be present sympatrically with *C. watsonii*-like ~2–6 μm spherical cells in particle export traps from the North Pacific Ocean over multiple years (Fig. 1E-G).

### Evolutionary relationships

**Figure 2:**
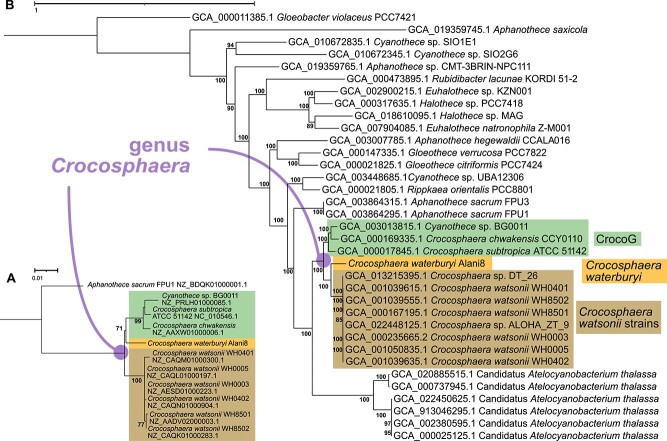
(**A**) The 16S rRNA gene tree of cultured *Crocosphaera*, and (**B**) the phylogenomic tree of 35 representative cyanobacterial taxa in order *Chroococcales* closely related to C. *waterburyi*. The CrocoG, C. *watsonii*, and C. *waterburyi* clades are labeled next to the tree. Bootstrap values below 70% are not shown for either tree. Tree scale is equal to 0.01 for (**A**) and 1 for (**B**).

A 16S rRNA phylogenetic tree was created using the genes from representative *Crocosphaera* isolates (Fig. 2A), and a phylogenomic tree was created with 350 genomes from NCBI assembly within the order *Chroococcales* and genus *Cyanothece* to ensure correct taxonomic placement of *C. waterburyi* (Supplemental Fig. S1). Following this, a subsequent tree was made using 35 representative, related taxa to *C. waterburyi* (Fig. 2B). At the 16S rRNA gene level, *C. waterburyi* represents a new species closest to the CrocoG (Fig. 2A). However, phylogenomically, *C. waterburyi* was more closely related to *C. watsonii* yet still clustered independently (Fig. 2B). *C. watsonii* and *C. waterburyi* also formed an “oceanic” phylogenomic group within the genus, which is distinct from the coastal CrocoG (Fig. 2B).

Different *C. watsonii* isolates have been shown to display strain-specific differences in cell size and exopolysaccharide (EPS) production [5, 63]. However, despite these differences, the *C. watsonii* strains were all phylogenomically closely related (Fig. 2). *C. waterburyi* displayed both morphological (Fig. 1) and strong phylogenetic differences from *C. watsonii* (Fig. 2A-B), in support of our proposal to describe it as a distinct species of *Crocosphaera*.

### Pangenomic comparisons of genus *Crocosphaera*

The full genomic potential and pangenomics of the genus *Crocosphaera* has never been characterized. Thus, how gene content varies across the genus, including *C. waterburyi*, has never been defined. To ensure that only high-quality genomes were included in the *Crocosphaera* pangenome, CheckM [64] was used to demonstrate that all genomes were > 98% complete, <2% contamination with N50 values between 9196 and 4 934 271 (Supplemental Table S1). The draft genome of *C. waterburyi*, specifically, was found to be high quality at 99.56% complete, 0% contamination, and an N50 of 16 538. The GC content of *C. waterburyi* (38.1%) was slightly higher than the *C. watsonii* strains (37.1–37.7%) but comparable to the coastal *Cyanothece* sp. BG0011 genome in the CrocoG subclade (38.2%).

**Figure 3:**
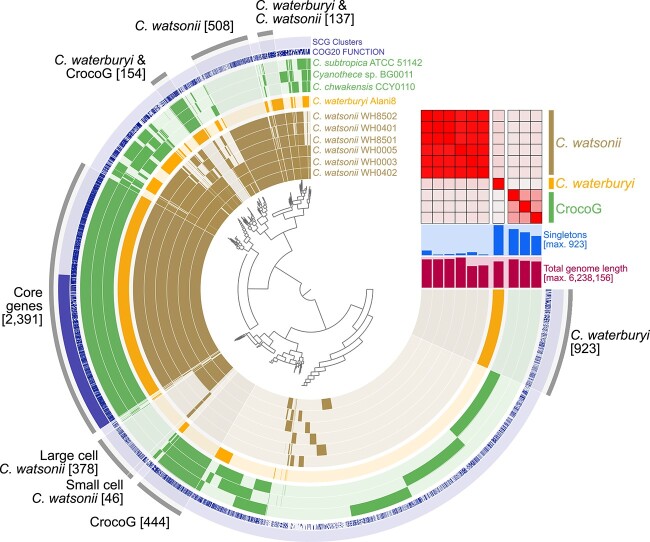
The pangenome of the genus *Crocosphaera*. The heatmap shows % ANI similarity and subclade distinctions of the genus with a lower threshold of 80% similarity, and the tree at the center shows gene cluster presence vs absence. Brackets indicate the number of gene clusters in each bin. The gene annotations are shown in the “COG20 function” layer, and the single copy genes in all 10 genomes are shown in the “SCG clusters” layer. The “singletons” (shown above “Total genome length”) are the number of gene clusters present only in individual genomes.

Members of the genus *Crocosphaera*, despite their wide biogeographical range and habitat difference (coastal vs oligotrophic), had 2391 gene clusters in their “genomic core,” (Fig. 3). The core genes were enriched in distinct functions related to the lifestyle of these organisms, including N_2_-fixation, phosphate uptake, iron (III) utilization, photosynthesis, phycobiliprotein, and mobile genetic element-related genes (Supplemental Table S2).

Pangenomic analysis also revealed that members of each phylogenomically-defined *Crocosphaera* clade had accessory genes found only in those groups. For example, CrocoG and *C. watsonii* subclades each had genes distinct to their groups (each group having 444 and 508 accessory gene clusters, respectively; Fig. 3), enriched in different mobile genetic element-related genes (Supplemental Table S2). *C. watsonii* also showed sub-grouping at the strain level with the small cell phenotypes having 46 specific accessory gene clusters in total and the large cell phenotype having 378 gene clusters (Fig. 3). Overall, *C. waterburyi* was found to have the largest set of unique genes with a total of 986 genes and 923 gene clusters (Fig. 3), although 51% lacked annotation by NCBI-COGS, Pfam, and KOfam. These high accessory gene numbers in *C. waterburyi* could be due to only one genome being available from this group. However, broad groupings based on the presence and absence of accessory genes corroborate the phylogenomic structure. *C. waterburyi* also shared distinct gene clusters with the CrocoG (154 gene clusters) and separately with *C. watsonii* strains (137 gene clusters), (Fig. 3; listed in Supplemental Table S2). Of particular interest were accessory genes found only in *C. waterburyi* and the CrocoG; this included *mreBCD* rod-shape determining proteins predicted to be responsible for the phenotypic difference in rod vs spherical shape of *C. waterburyi* and the CrocoG vs *C. watsonii* cells. These genes were confirmatory that the rod shape observed in the CrocoG and *C. waterburyi* is a true evolutionary difference from *C. watsonii*.

When further visualized and compared by average nucleotide identity (ANI), (>80% lower threshold), *Crocosphaera* were again differentiated into the same three subclades: *C. watsonii* strains, the CrocoG, and *C. waterburyi*. As expected, the six *C. watsonii* genomes had high ANI identity at >98%. However, *C. waterburyi* was only 82% ANI to all cultured *C. watsonii* strains and 80–81% to the CrocoG (Supplemental Table S3). As these values are below both the suggested intra-species 95% ANI cutoff and the 83% ANI inter-species value [43], this supports the species designation of *C. waterburyi*. In summary, based on both gene content and % ANI, *C. waterburyi* shares features with both the green, coastal, and orange, oligotrophic *Crocosphaera* subclades.

Although *C. waterburyi* and *C. watsonii* have specific conserved genes (Fig. 3) and similar habitats, there are unique genetic characteristics of each. One prime example was the presence of a CRISPR-Cas type I-B system in *C. waterburyi* (Supplemental Figs. S2-S3, Supplemental Table S4) but not in any of the six *C. watsonii* strains. The *C. watsonii* strains all encoded only Csa3, which was annotated as a transposase and not a true Cas gene [65]. CRISPR-cas systems can provide bacteria with immunity against bacteriophage infection [66], and cyanobacteria frequently have the Type III-B system [67], including the sympatric cyanobacterium *Trichodesmium thiebautii* [65]*.* However, based on analyses with CCTyper [68] and Anvi’o [36], *C. waterburyi* and other closely related single-celled cyanobacteria encode the Type I-B system (Supplemental Figs. S2-S3). With this I-B CRISPR-cas system, *C. waterburyi* may be more resistant to cyanophage infection than *C. watsonii*. However, isolation of more *C. waterburyi* strains and additional environmental sequencing efforts are needed to address this further.

Although several Fe (III) and (II) utilization genes (*feoAB*, *afuA*, *fbpB*) were shared by all *Crocosphaera* genomes, accessory Fe (II) utilization *feoAB* genes were found to vary between *C. waterburyi*, *C. watsonii,* and CrocoG genomes (Fig. 3; **Core genes**; Supplemental Table S2). This finding is relevant as Fe demand is increased in oligotrophic ocean diazotrophs relative to other phytoplankton due to their obligatory Fe requirement of the metalloenzyme nitrogenase [1]. For example, *C. waterburyi* was found to encode a second additional Fe (II) transporter via the maintenance of distinct *feoAB* genes (Supplemental Table S2, S5). Blastp identified them as more similar by % identity to *feoAB* in *Gloeocapsa* sp. PCC 73106 (WP_006528539.1, WP_006528538.1), which are of freshwater origin [69]. This implied a hereditary difference and potential horizontal gene transfer event. Fe (II) is not common in oxygenated seawater, but its transport genes were conserved in other “aggregating” oceanic diazotrophs [70, 71]. Therefore, it is possible that these extra transporters are important in *C. waterburyi* aggregates wherein O_2_ is likely reduced nightly due to respiration.

In summary, *Crocosphaera*, including *C. waterburyi*, are overall similar in GC %, genome size, and core metabolic features. However, distinct genetic functions, such as differences in Fe utilization genes and predicted phage immunity, distinguish the oceanic species, *C. watsonii* and *C. waterburyi,* and inform on their individual ecological roles.

### *Crocosphaera* biogeography in the oligotrophic oceans

The Earth’s oligotrophic oceans are characterized as low-nutrient, high microbial remineralization regions, and unlike the coastal ocean, these oceanographic “deserts” are vast in size, comprising >60% of the global oceans [72]. Organisms in these ecosystems rely heavily on N_2_ fixation by diazotrophs, including *Crocosphaera*, in the euphotic zone to fuel microbial to upper trophic level productivity [1, 2, 8]. Therefore, determining where oligotrophic *Crocosphaera* species are present and active is important for understanding their contributions to global biogeochemistry.

**Figure 4:**
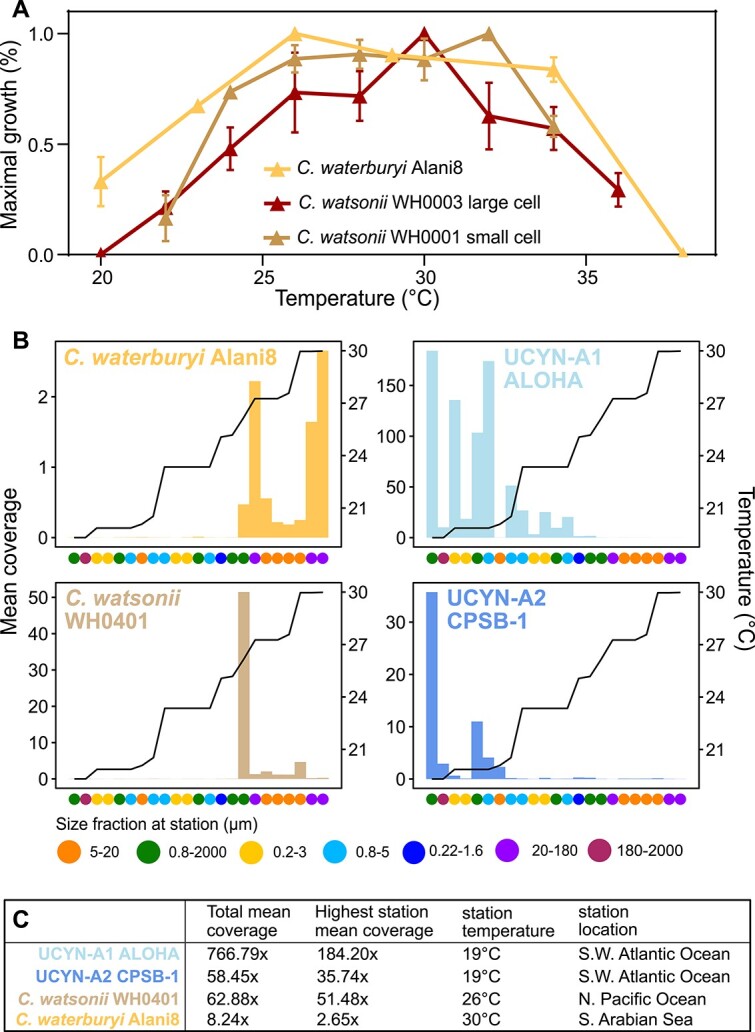
Thermal optima of *C. watsonii* strains and *C. waterburyi* Alani8 in culture conditions (**A**) and extrapolated from environmental metagenomes (**B-C**). In (**A**), growth rates are normalized to % maximal growth for each temperature and strain, and error bars show standard error. The mean coverage values (left y-axis) across TaraOceans samples for representative marine unicellular diazotroph strains are shown in (**B**). In (B), dots on the x-axis indicate all sample size fractions, samples are ordered by increasing temperature, and the temperature at each station was overlayed as a black line. The right y-axis shows the temperature scale. In (**C**), the following are shown from left to right: Total mean coverages for each genome across all stations, the individual station where each genome had the highest mean coverage (was most abundant), the station temperature where each genome had the highest mean coverage, and the station location.

*C. waterburyi* and *C. watsonii* were demonstrated to have morphological and genomic similarities and differences (Figs. 1–3)*,* so culturing experiments were carried out to compare their physiologies. *C. waterburyi* Alani8 and *C. watsonii* WH0003 cultures grown at 26°C and ~150 μmol Q m^−2^ s^−1^ were found to have similar growth rates and N_2_ fixation under these conditions, and they both fixed N_2_ at night (Supplemental Fig. S4). Following this, replicate cultures of *C. waterburyi* Alani8 were grown from 20–38°C at 96 μmol Q m^−2^ s^−1^ in a 12:12 light: dark cycle to determine its full thermal growth range. These values were compared to those previously recorded for multiple *C. watsonii* strains [5]. From this comparison, it was found that *C. waterburyi* Alani8 had a wide thermal optimum (23–34°C), and its growth at 34°C exceeded that of the two representative large and small cell *C. watsonii* strains (Fig. 4A; Supplemental Table S6).

To further explore these differences in an ecological context, genomes from the oligotrophic marine unicellular cyanobacterial diazotrophs, including both *Crocosphaera* species and the closely-related cyanobacterial endosymbiont UCYN-A [73], were used to recruit reads from 934 TaraOceans metagenomes (stations listed in Supplemental Table S7A-B). The surface stations where ≥1 unicellular diazotroph was present at >1x mean coverage was compared to sampling station temperatures (Fig. 4B–C). *C. waterburyi* Alani8 had the highest mean coverage at a 29.98°C station in the Arabian Sea whereas *C. watsonii* WH0401 had the highest mean coverage at a 26.17°C station in the North Pacific Ocean (Fig. 4B–C). UCYN-A strains had the highest mean coverage at 19°C in the South-West Atlantic Ocean (Fig. 4B–C). In addition to TaraOceans, other metagenomes from BioGEOTRACES and GO-SHIP, were read recruited to *C. watsonii*, CrocoG, and *C. waterburyi* Alani8 genomes. *C. watsonii* WH0003 was present at >25% genome detection in a small number of samples from BioGEOTRACES and GO-SHIP, but *C. waterburyi* and the CrocoG were absent (Supplemental Table S7A). Together, these physiological and environmental data imply that *C. watsonii* and UCYN-A are more successful under modern ocean conditions and have lower thermal optima than *C. waterburyi* in culture and the ocean. However, if oligotrophic gyre temperatures rise consistently over 30°C during climate change, *C. waterburyi* may become more abundant in the unicellular cyanobacteria community and extend its biogeographical range.

*C. watsonii* distribution and abundance has been previously well characterized in the North Pacific Ocean near Station ALOHA [3, 9, 74], and they have been observed as consistent members of the bacterial community, particularly during the summer. However, despite being isolated from the North Pacific Ocean near Station ALOHA, the abundance and activity of *C. waterburyi* were previously uncharacterized in this region.

**Figure 5:**
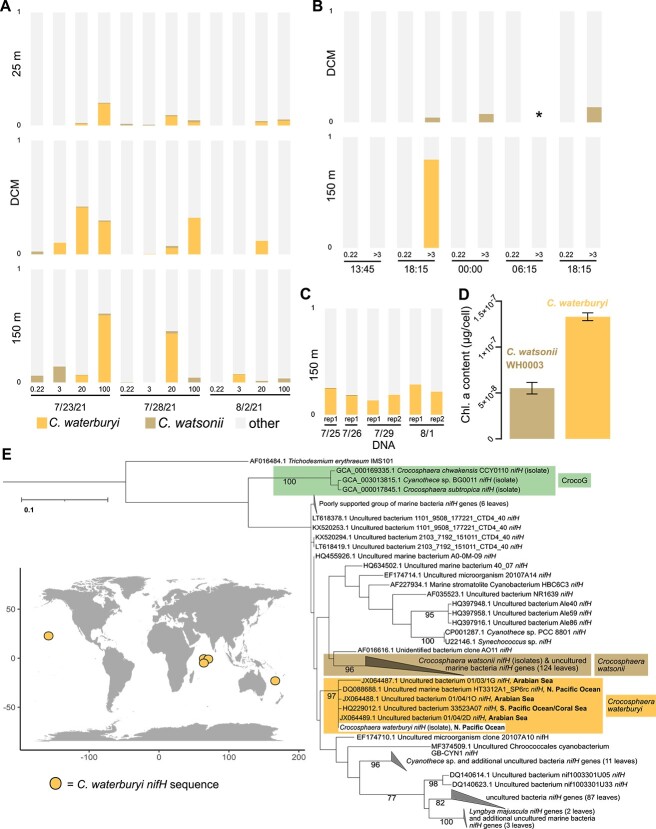
The *nifH* gene relative of abundance of *C. waterburyi*, *C. watsonii*, and other diazotrophs in the North Pacific Ocean. Shown are the size-fractionated *nifH* gene relative abundance from deployed net traps (**A**), *nifH* transcripts from a diel sampling (**B**), and the *nifH* gene presence over 4 days in 150 m net traps (**C**). The DCM fell at a depth of 135 m, and data was not available for one DCM >3-μm size fraction sample over the diel sampling (marked with an “*”). The low light grown (~30 μmol Q m^−2^ s^−1^) chlorophyll a cell^−1^ for *C. watsonii* WH0003 and *C. waterburyi* Alani8 is shown, and error bars indicate standard error (**D**). The *nifH* DNA phylogeny of 250 NCBI-blastn hits closest to *C. waterburyi* and the locations where the sequences originated are shown in (**E**). For the world map in (**E**), the three dots indicating sequences from the Arabian Sea are overlapping in coordinate and are very slightly offset in the map from their actual coordinates. All exact coordinates are recorded in Supplemental Table S8.

To determine *C. waterburyi* relative abundance in the North Pacific, we utilized a summer 2021 diel *nifH* amplicon DNA/RNA dataset collected from the surface, DCM, and 150 m particle traps in the Station ALOHA region. This showed that the *C. waterburyi nifH* gene had highest relative abundances, particularly in the 20 and 100 μm size fractions, at the DCM, and in 150 m depth samples (Fig. 5A–C). As *C. waterburyi* cells are only ~5 μm long (Fig. 1), their presence in larger size fractions (20 and 100 μm) provides evidence that these cells likely form large aggregates *in situ*, as has been observed in the TaraOceans metagenomes and in culture with the Alani8 strain (Fig. 1).

Transcripts 100% identical to *C. waterburyi nifH* were detected in the early evening (18:15) in the 3-μm size fraction at 150 m depth (Fig. 5B). However, contrastingly, *C. watsonii nifH* transcripts were found at the DCM (130 m), (Fig. 5B). *C. waterburyi* also had a 100% identity match to the uncultivated “Croco_otu3,” recently sequenced from the North Pacific, which had higher relative abundance deeper in the euphotic zone (150 m) over ~3 years of sampling [75]. These findings suggest a potential difference in how deep in the water column these species can exist and remain active. To explore this with cultures, *C. waterburyi* and *C. watsonii* WH0003 were grown under low light (30 μmol Q m^−2^ s^−1^) approximating the base of the euphotic zone near the DCM or directly below. Under these conditions, *C. waterburyi* had ~2x the amount of chlorophyll a cell^−1^ as *C. watsonii* (Fig. 5D), providing a potential mechanism through which *C. waterburyi* can remain active deeper in the water column than *C. watsonii*. However, further experiments and characterization of multiple strains are needed to explore this trend in more detail.

In addition to these recent datasets, we analyzed historical *nifH* amplicon data using blastn and the *C. waterburyi* isolate *nifH* gene to determine presence in the North Pacific (*nifH* = 80–80.5% to the CrocoG and 93.4–93.7% identity to *C. watsonii* strains). The top 250 sequences from blastn were then aligned and phylogenetically compared. The *C. waterburyi* Alani8 *nifH* gene clustered with a *nifH* sequence from the North Pacific Ocean as well as the South Pacific/Coral Sea (Fig. 5E; Supplemental Table S8). Additionally, the *C. waterburyi* isolate *nifH* sequence matched at 100% identity to a *nifH* amplicon (Fig. 5E) sequenced from the Arabian Sea [76], which aligned well with the TaraOceans biogeography trend (Fig. 4B–C). Overall, these data support *C. waterburyi*’s presence in the global oceans.

**Figure 6:**
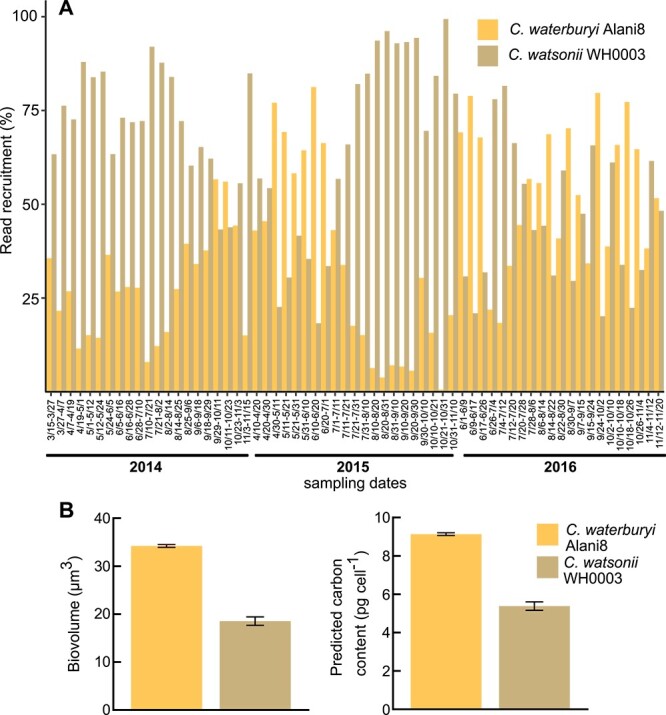
Read mapping of *C. waterburyi* and *C. watsonii* WH0003 genomes to 4000 m sediment trap metagenomic samples from 2014–2016. The % recruitment of mapped reads is shown for *C. watsonii* and *C. waterburyi* (**A**), (interpretation: Of the reads that were mapped, X% mapped to *C. watsonii* and X% mapped to *C. waterburyi*). The CrocoG were included in the analysis but are not shown here as their % genome detection across all samples was always <0.4%. In (**B**), the biovolume and calculated carbon content for representative strains of both oligotrophic *Crocosphaera* species are shown, and error bars indicate standard error.

Microscopic data in Fig. 1, showed that rod-shaped *C. waterburyi-*like cells were found in particle traps in 2010 and 2021, and Station ALOHA *nifH* data showed that *C. waterburyi* was present and active in the North Pacific (Fig. 1; Fig. 5A–E). Together, these data suggest that *C. waterburyi* is a contributor to C + N export in the North Pacific either through sinking or in zooplankton fecal pellets. To test this further, the *C. waterburyi*, *C. watsonii*, and CrocoG genomes were used to recruit reads from Station ALOHA, North Pacific 4000 m deep trap metagenomic samples, which had been previously used to assemble and read recruit to a *C. watsonii*-like environmental MAG [11, 12, 14, 38, 44]. This effort showed that *C. waterburyi* and *C. watsonii* were detected at >25% genome presence across all three years (2014–2016), whereas the CrocoG were not (Fig. 6; Supplemental Table S7A). However, *C. watsonii* and *C. waterburyi* had different % recruitment values across these years, with *C. waterburyi* increasing in % recruitment from 2014 to 2016 and becoming relatively more abundant across seasons in 2016 (Fig. 6).

Since both *C. waterburyi* and *C. watsonii* were found to be contributors to C + N export, the biovolume of individual cells were measured in cultures grown at low light. These conditions were chosen to simulate where *Crocosphaera* species were transcriptionally active (130–150 m) but likely sinking out. *C. waterburyi* was found to have ~2x the biovolume and predicted carbon content as *C. watsonii* WH0003 under these growth conditions (Fig. 6B). Media type, light intensity, and temperature can have an effect on cell size differences in *C. watsonii* [5, 77]. However, generally, during the years that capsule-shaped *C. waterburyi* Alani8 was more abundant in 4000 m sediment traps, there may have been increased C + N export from genus *Crocosphaera* overall. Further work on *C. waterburyi* abundances on sinking particles will tease apart C + N export dynamics of this species; this is of particular interest as C fixation and export by photosynthetic organisms have implications for deep ocean carbon sequestration.

### Taxonomic appendix

*Crocosphaera waterburyi* C.S. Cleveland et E.A. Webb, nov. sp.

Figures 1–6; S1–S4.

*Diagnosis*: The single unicells are shorter capsules when recently divided and elongate when preparing to divide. The cellular shape contrasts with the closest known species, *Crocosphaera watsonii*, which are spherical in shape.

*Description*: The single unicells appear orange under DAPI-LP excitation, which indicates a phycoerythrin-rich pigmentation. Unicells can become embedded in layers of EPSs excreted by the cells and can form aggregates of 50–100 cells (Fig. 1A). Individual unicells are 4–6 μm in length by 2–3 μm wide. Cells can be seen adhering to sides of culture flasks but can be generally removed back into solution by gentle agitation. Within ~2–5 days after transfers, liquid cultures will take on orange pigmentation, and culture solutions will become highly viscous. When phylogenetically compared to other cultured *Crocosphaera*, the 16S rRNA gene clustered in a distinct subclade separate from other species. The genome has *nif* genes, *nifH,* which is expressed in the North Pacific Ocean (Fig. 4) and fixes atmospheric nitrogen in culture. The genome also encodes genes for phycobilisome assembly, photosynthesis, and carbon fixation. Overall health of cultures can be assessed using DAPI-LP epifluorescence microscopy; dead or dying cells will appear light green or light blue and healthy cells will still be orange in color.

*Habitat*: Pelagic oligotrophic oceans at 0–150 m depth.

*Type locality*: Station ALOHA, North Pacific Ocean.

*Holotype*: Alani8 strain, dried and preserved biomass deposited at University of California Berkeley Herbarium under accession number UC2110199, live cultures maintained at the NCMA at Bigelow under accession number CCMP 3753.

*Reference strain*: *Crocosphaera waterburyi* Alani8.

*Etymology*: *Crocosphaera*, Gr. masc. n. krokos, crocus, orange colored; Gr. fem. n. sphaîra, ball or sphere; species *waterburyi* after John Waterbury, who discovered *C. watsonii*.

## Conclusion

*Crocosphaera* are keystone species in the marine food web that bring new sources of organic C + N into low nutrient, oligotrophic ocean regions [2, 4, 5, 9]. In a changing global climate, understanding these important links in marine microbial communities is essential for predicting environmental outcomes. Despite being sympatric in ocean gyres, *C. waterburyi* has larger cellular biovolume than *C. watsonii* in low light conditions due to its rod shape, and therefore, may be more impactful on C + N export than some *Crocosphaera* phenotypes in the North Pacific Ocean. Also, the *C. waterburyi* culture was found to grow better at high temperatures than *C. watsonii,* and environmental genomic read-mapping data corroborated this. These data suggest that *C. waterburyi* prefers warmer surface waters.

The discovery of *C. waterburyi* demonstrates that there is still more to be learned about oceanic N_2_-fixer diversity. This study also highlights the need for more isolation efforts of *C. waterburyi* strains and qPCR surveys to determine their absolute abundance. As well, it warrants further studies focused broadly on the genus *Crocosphaera*, both in sinking particles and the surface ocean, to understand how they may respond and change under anthropogenic warming of the oceans.

## Supplementary Material

SUPPLEMENTAL_111824_wrae217

supplemental_tables_111624_wrae217

Figure_S1_wrae217

Figure_S2_wrae217

Figure_S3_wrae217

Figure_S4_wrae217

## Data Availability

The whole genome sequence of *C. waterburyi* Alani8 has been deposited on GenBank under BioProject PRJNA951741 and BioSample SAMN34055600. The raw forward and reverse reads are available on the NCBI Sequence Read Archive project PRJNA951741. Demultiplexed raw *nifH* amplicon sequences are available on the NCBI Sequence Read Archive under BioProject PRJNA1009239. Code for bioinformatic pipelines can be found at: https://github.com/catiecleveland/Crocosphaera-Biogeography.
