# Supplementary material for: Isolation and biogeography of the oligotrophic ocean diazotroph, Crocosphaera waterburyi nov. sp
Source: ISME J. 2024 Oct 23;18(1):wrae217. doi: 10.1093/ismejo/wrae217 (PMC11630315; doi:10.1093/ismejo/wrae217)
Supplement: SUPPLEMENTAL_111824_wrae217 [file supplemental_111824_wrae217.docx]

**Isolation and biogeography of the oligotrophic ocean diazotroph, *Crocosphaera waterburyi* nov. sp.**

Catie S. Cleveland, Kendra A. Turk-Kubo, Yiming Zhao, Jonathan P. Zehr, Eric A. Webb*

**Supplemental Figures S1-S4**

**Supplemental Methods and References**

**Supplemental Methods**

**Microscopy**

Scanning electron microscopy (SEM) images were taken on the Apreo 2 SEM microscope (Thermo Fisher, Waltham, MA, USA). Prior to imaging, the sample was fixed in 2.5% glutaraldehyde, filtered onto 0.8 µm filters, and microwaved for 4x in 1 minute on/off intervals at 150 watts 2x and 250 watts 2x. The sample was then washed 1x in 0.1M HEPES with 5% sucrose and 2x in ultra-pure water with alternating 1 minute microwave steps at 250 watts. Samples were then dehydrated using serial EtOH washes starting at 50% EtOH and up to 100% with 1 minute of 250 watts microwaving at each step. The last step with 100% EtOH was done with molecular sieves. The sample was further dried in critical point drier (Tousimis, Rockville, MD, USA), and were briefly coated with a carbon coater (Cressington, Watford, UK) prior to imaging.

**Genome Assembly**

The assembly pipeline is briefly summarized as follows: FastQC v0.11.9 checked read quality [1], Trimmomatic v0.36 removed contaminating and low scoring sequences with a sliding window size of 4 and minimum quality of 15 (0.1% reads dropped) [2], Kaiju Taxonomic Classifier v1.5.0 described the taxonomic distribution of the reads [3], MetaSPAdes v3.13.0 assembled the reads with default parameters [4], MaxBin2 v2.2.4 binned the contigs to obtain metagenome assembled genomes (MAGs), [5], CheckM v1.0.18 analyzed quality [6], and GTDB-tk v1.7.0 [7] determined the taxonomy of *C. waterburyi.*

**Chlorophyll and Biovolume Estimates**

*C. watsonii* and *C. waterburyi* cultures were also grown under low light (26°C, 12:12 diel cycle, 30 µmol m^-2^ s^-1^), which simulated the deep euphotic zone (130-150 m), for >1 month. Cultures were transferred at 1:3 dilution 2 days prior to sampling to avoid nutrient limitation effects. Following this, cultures were thoroughly mixed, and cell counts were taken with a hemacytometer, 5 mL of cultures were filtered onto 0.7 µm Whatman® GFF filters, chlorophyll a was extracted at 4°C from filters in 90% acetone overnight as per previous methods [8] and quantified on a Trilogy fluorometer (Turner Designs, San Jose, CA, USA) with a non-acidified module to yield chlorophyll a values in µg cell^-1^.

Photos of the same low light *C. watsonii* WH0003 and *C. waterburyi* Alani8 (grown at 26°C, 12:12 diel cycle, 30 µmol m^-2^ s^-1^) were also taken using bright field light, imported into ImageJ, and cell sizes were measured using the “edge detection” feature to calculate intracellular biovolume and carbon content. *C. watsonii* WH0003 and *C. waterburyi* Alani8 biovolumes were calculated as spheres and capsules, respectively. Carbon content was extrapolated from biovolume using a previously-defined equation [9, 10].

**Growth Rate and N_2_-fixation Comparisons**

N_2_ fixation of *C. waterburyi* Alani8 and *C. watsonii* WH0003 were measured in triplicate using the acetylene reduction assay [11]. Triplicate Nalgene 43 mL polycarbonate tubes were used and contained 30 mL of each culture and 13 mL of headspace. The tubes were sealed with tightened septa caps (I-CHEM, Calhoun, LA, USA), charged with >10% acetylene synthesized from carbide (Thermo Fisher Scientific Inc., Waltham, MA, USA), and produced ethylene was measured on a GC-8a gas chromatograph (Shimadzu, Kyoto, Japan) following 12 hours incubation. Cultures were incubated under the following conditions: cool white lights, ~150 µmol m^-2^ s^-1^, 12:12 diel cycle, and 26°C. Acetylene reduction was calculated using 9 ppm ethylene standards (Cambridge Isotopes, Cambridge, MA, USA). Final rates were normalized to raw in-vivo chlorophyll a fluorescence.

Growth rates of *C. watsonii* and *C. waterburyi* were determined from triplicate in-vivo chlorophyll fluorescence data obtained on a TD-700 Fluorometer (Turner Designs, San Jose, CA, USA). Cultures were grown under the same conditions as previously described for N_2_ fixation. Before each measurement, cultures were thoroughly mixed to fully homogenize aggregates in solution. Growth rates were calculated using the raw fluorescence increase and the Malthusian exponential growth model in GraphPad Prism v9 (La Jolla, CA, USA).

For thermal profile growth rate comparisons using *C. waterburyi* growth data and previously collected data [12], the % maximal growth was calculated by dividing growth rates across temperatures by the fastest growth rate exhibited per replicate. For example, if the growth rate in each strain for a replicate was lowest at 0.1 days^-1^ at 20°C and highest at 0.4 days^-1^ at 34°C, then 34°C would be 100% of maximal growth and 20°C would be 25% of the maximal growth possible for a given replicate set in each strain.

**CRISPR-cas Annotation**

All GenBank genomes in the phylogenomic tree (**Figure 2**) were downloaded from NCBI and annotated with CCTyper [13]. Organisms found to have some or all non-putative Cas genes necessary for a CRISPR-cas system were identified and superimposed on the phylogenomic tree. One CRISPR-cas system annotated as “ambiguous” was not included. Further descriptions of CCTyper are found at: https://github.com/Russel88/CRISPRCasTyper.

**CRISPR-cas 8b Gene Phylogenetics**

Seven organisms in the representative phylogenomic tree within families *Aphanothecaceae*/*Microcystaceae*, when annotated by CCTyper [13], were shown to encode a Type I-B CRISPR-cas system with a full length Cas8 gene. CCTyper provided color coding for different types of genes in the CRISPR-cas systems. For each of these seven organisms, a contigs database was generated using Anvi’o v7.1 [14]. The contigs databases were visualized and subsequently the Cas8b diagnostic gene for the Type I-B system [15] were found and aligned with using Clustal Omega 1.2.2 [16], trimmed, and a Cas8b gene tree was created using RAxML 8.2.11 [17] with a GTR GAMMA nucleotide model, rapid bootstrapping (1000 bootstraps), and a search for best-scoring maximum likelihood tree algorithm.

**References**

1. Andrews S. FastQC: a quality control tool for high throughput sequence data. Babraham Bioinformatics, Babraham Institute, Cambridge, United Kingdom 2010.

2. Bolger AM, Lohse M, Usadel B. Trimmomatic: a flexible trimmer for Illumina sequence data. *Bioinformatics* 2014; **30**: 2114–2120.

3. Menzel P, Ng KL, Krogh A. Fast and sensitive taxonomic classification for metagenomics with Kaiju. *Nat Commun* 2016; **7**: 11257.

4. Nurk S, Meleshko D, Korobeynikov A, Pevzner PA. metaSPAdes: a new versatile metagenomic assembler. *Genome Res* 2017; **27**: 824–834.

5. Wu Y-W, Simmons BA, Singer SW. MaxBin 2.0: an automated binning algorithm to recover genomes from multiple metagenomic datasets. *Bioinformatics* 2016; **32**: 605–607.

6. Parks DH, Imelfort M, Skennerton CT, Hugenholtz P, Tyson GW. CheckM: assessing the quality of microbial genomes recovered from isolates, single cells, and metagenomes. *Genome Res* 2015; **25**: 1043–1055.

7. Chaumeil P-A, Mussig AJ, Hugenholtz P, Parks DH. GTDB-Tk: a toolkit to classify genomes with the Genome Taxonomy Database. *Bioinformatics* 2019; **36**: 1925–1927.

8. Strickland JDH, Parsons TR. A practical handbook of seawater analysis. *Fisheries Research Board of Canada* 1972: 185-206.

9. Verity PG, Robertson CY, Tronzo CR, Andrews MG, Nelson JR, Sieracki ME. Relationships between cell volume and the carbon and nitrogen content of marine photosynthetic nanoplankton. *Limnol Oceanogr* 1992; **37**: 1434–1446.

10. Luo Y-W, Doney SC, Anderson LA, Benavides M, Berman-Frank I, Bode A, et al. Database of diazotrophs in global ocean: abundance, biomass and nitrogen fixation rates. *Earth Syst Sci Data* 2012; **4**: 47–73.

11. Capone DG. Determination of nitrogenase activity in aquatic samples using the acetylene reduction procedure. *Handbook of methods in aquatic microbial ecology* 1993: 621-631.

12. Webb EA, Ehrenreich IM, Brown SL, Valois FW, Waterbury JB. Phenotypic and genotypic characterization of multiple strains of the diazotrophic cyanobacterium, *Crocosphaera watsonii*, isolated from the open ocean. *Environ Microbiol* 2009; **11**: 338–348.

13. Russel J, Pinilla-Redondo R, Mayo-Muñoz D, Shah SA, Sørensen SJ. CRISPRCasTyper: Automated Identification, Annotation, and Classification of CRISPR-Cas Loci. *CRISPR J* 2020; **3**: 462–469.

14. Eren AM, Kiefl E, Shaiber A, Veseli I, Miller SE, Schechter MS, et al. Community-led, integrated, reproducible multi-omics with anvi’o. *Nat Microbiol* 2021; **6**: 3–6.

15. Makarova KS, Wolf YI, Iranzo J, Shmakov SA, Alkhnbashi OS, Brouns SJJ, et al. Evolutionary classification of CRISPR-Cas systems: a burst of class 2 and derived variants. *Nat Rev Microbiol* 2020; **18**: 67–83.

16. Sievers F, Wilm A, Dineen D, Gibson TJ, Karplus K, Li W, et al. Fast, scalable generation of high-quality protein multiple sequence alignments using Clustal Omega. *Mol Syst Biol* 2011; **7**: 539.

17. Stamatakis A. RAxML version 8: a tool for phylogenetic analysis and post-analysis of large phylogenies. *Bioinformatics* 2014; **30**: 1312–1313.

**S1.** A phylogenomic tree created with GToTree using 350 genomes from order Chroococcales (including *Microcystaceae* and *Aphanothecaceae*). Bootstraps above 75% are shown. The tree scale is equal to 1.

**S2.** CRISPR-cas annotation of 35 unicellular N_2_-fixing cyanobacteria **(A)** and the specific cas genes and CRISPR arrays in genus *Crocosphaera* **(B)**. *C. waterburyi* and the CrocoG encode cas genes, CRISPR arrays, and spacer sequences. In **(B)**, yellow shows the interference module, blue shows the adaptation module, purple shows the accessory genes, and red shows Cas6 (color code provided by CCTyper).

**S3.** The Cas8b DNA sequences (diagnostic gene for Type I-B system) from the organisms in the representative phylogenomic tree within family *Aphanothecaceae*/*Microcystaceae* that encoded a Type I-B CRISPR-cas system and full Cas8b gene compiled into a phylogenetic tree. Only bootstraps above 75% are shown. The tree scale is equal to 1.

**S4.** The log growth of *C. waterburyi* Alani8 and *C. watsonii* WH0003 grown at 26°C **(A)**, and the nighttime N_2_ fixation at 26°C (**B**).
