# Supplementary figures and images for: Isolation and biogeography of the oligotrophic ocean diazotroph, Crocosphaera waterburyi nov. sp
Source: ISME J. 2024 Oct 23;18(1):wrae217. doi: 10.1093/ismejo/wrae217 (PMC11630315; doi:10.1093/ismejo/wrae217)

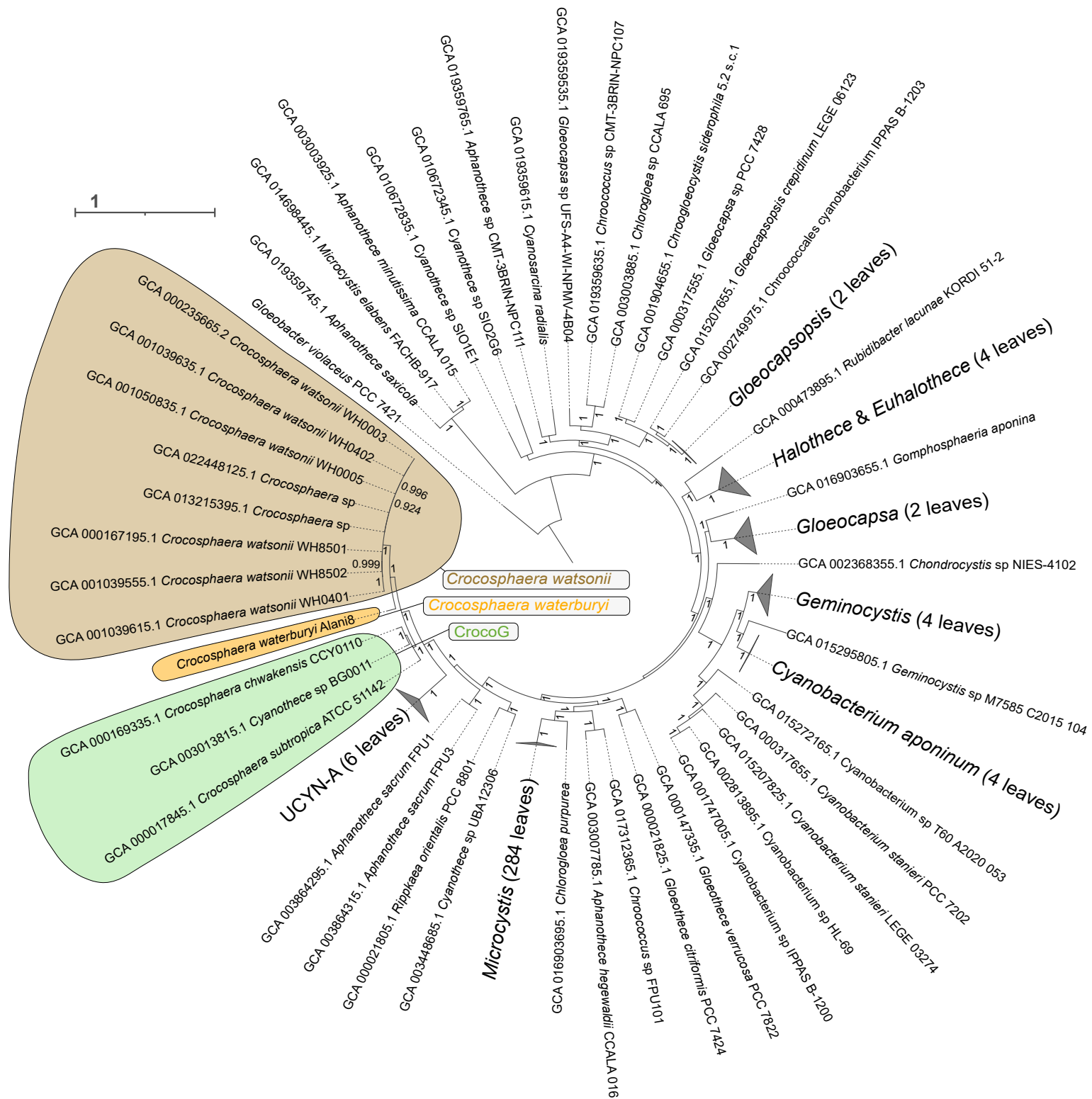

Supplement: Figure_S1_wrae217 [file figure_s1_wrae217.pdf]

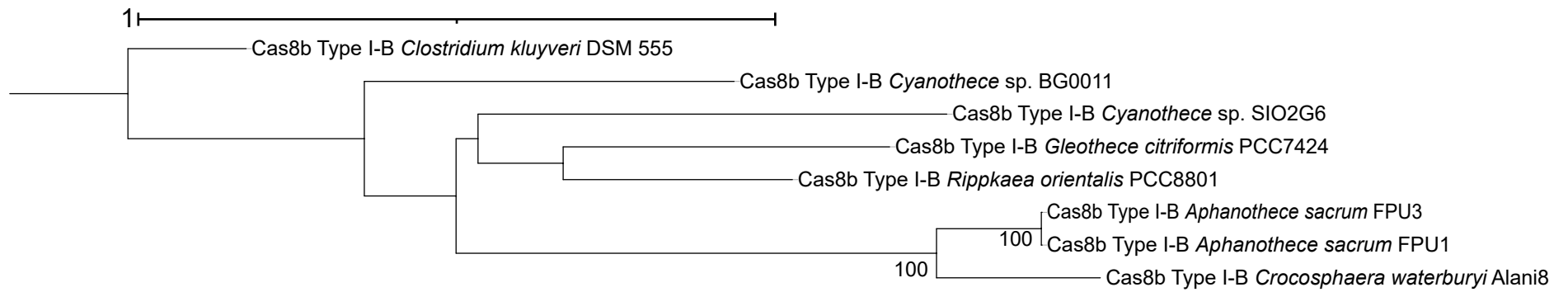

Supplement: Figure_S3_wrae217 [file figure_s3_wrae217.pdf]

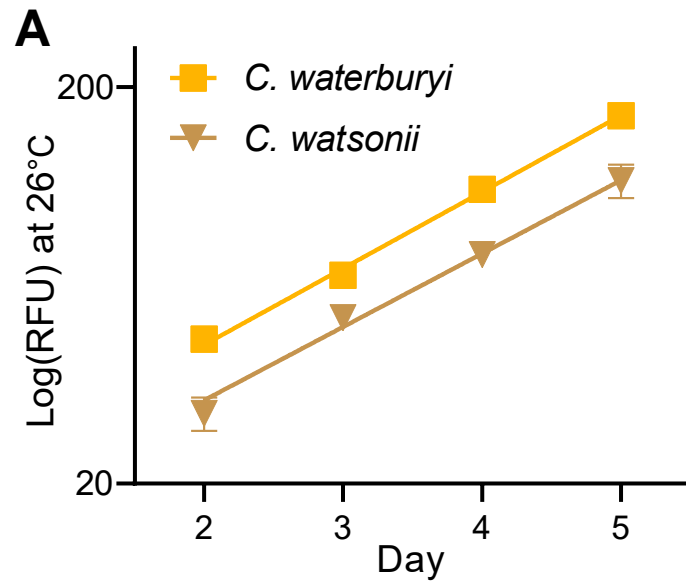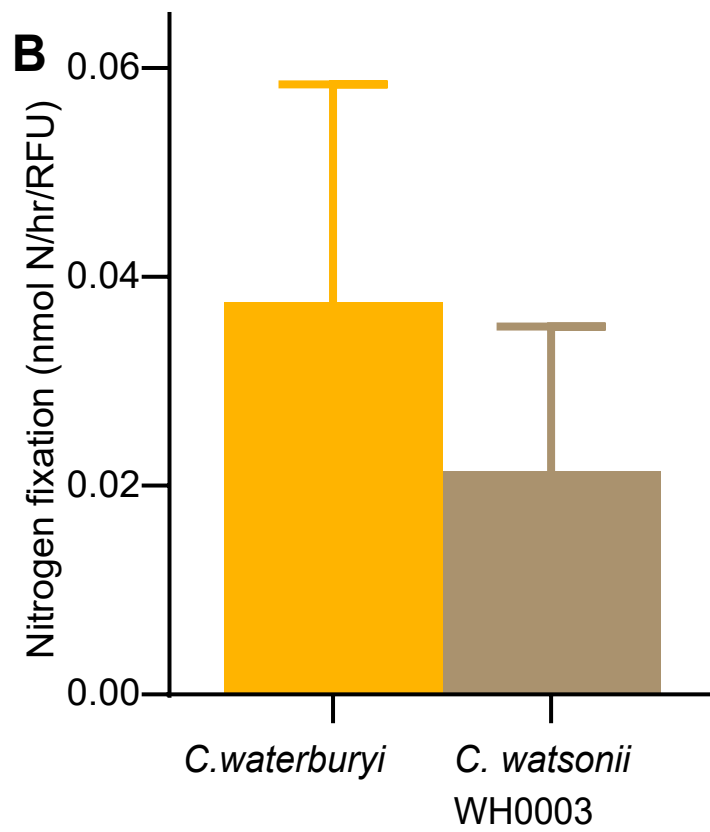

Supplement: Figure_S4_wrae217 [file figure_s4_wrae217.pdf]
