# Supplementary material for: Isolation and biogeography of the oligotrophic ocean diazotroph, Crocosphaera waterburyi nov. sp
Source: ISME J. 2024 Oct 23;18(1):wrae217. doi: 10.1093/ismejo/wrae217 (PMC11630315; doi:10.1093/ismejo/wrae217)
Supplement: Figure_S2_wrae217 [file figure_s2_wrae217.pdf]

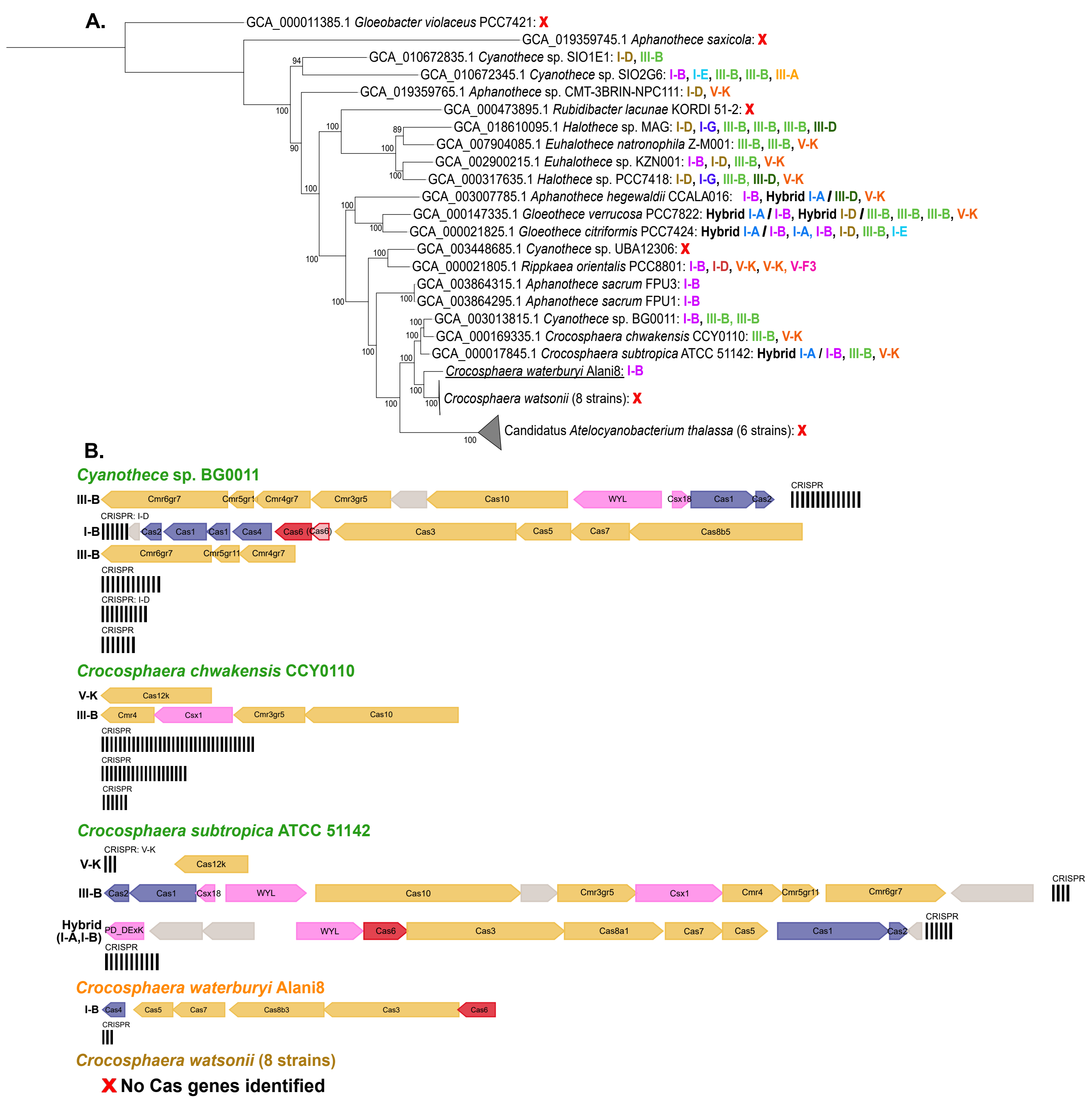
A.
GCA_000011385.1 Gloeobacter violaceus PCC7421: X
GCA_019359745.1 Aphanothece saxicola: X
GCA_010672835.1 Cyanothece sp. SIO1E1: I-D, III-B
GCA_010672345.1 Cyanothece sp. SIO2G6: I-B, I-E, III-B, III-B, III-A
GCA_019359765.1 Aphanothece sp. CMT-3BRIN-NPC111: I-D, V-K
GCA_000473895.1 Rubidibacter lacunae KORDI 51-2: X
GCA_018610095.1 Halothece sp. MAG: I-D, I-G, III-B, III-B, III-B, III-D
GCA_007904085.1 Euhalothece natronophila Z-M001: III-B, III-B, V-K
GCA_002900215.1 Euhalothece sp. KZN001: I-B, I-D, III-B, V-K
GCA_000317635.1 Halothece sp. PCC7418: I-D, I-G, III-B, III-D, V-K
GCA_003007785.1 Aphanothece hegewaldii CCALA016: I-B, Hybrid I-A / III-D, V-K
GCA_000147335.1 Gloeothece verrucosa PCC7822: Hybrid I-A / I-B, Hybrid I-D / III-B, III-B, III-B, V-K
GCA_000021825.1 Gloeothece citriformis PCC7424: Hybrid I-A / I-B, I-A, I-B, I-D, III-B, I-E
GCA_003448685.1 Cyanothece sp. UBA12306: X
GCA_000021805.1 Rippkaea orientalis PCC8801: I-B, I-D, V-K, V-K, V-F3
GCA_003864315.1 Aphanothece sacrum FPU3: I-B
GCA_003864295.1 Aphanothece sacrum FPU1: I-B
GCA_003013815.1 Cyanothece sp. BG0011: I-B, III-B, III-B
GCA_000169335.1 Crocosphaera chwakensis CCY0110: III-B, V-K
GCA_000017845.1 Crocosphaera subtropica ATCC 51142: Hybrid I-A / I-B, III-B, V-K
Crocosphaera waterburyi Alani8: I-B
Crocosphaera watsonii (8 strains): X
Candidatus Atelocyanobacterium thalassa (6 strains): X
94
100
90
100
89
100
100
100
100
100
100
100
100
100
100
100
100
100
100
100
100
100
B.
Cyanothece sp. BG0011
III-B
Cmr6gr7
Cmr5gr11
Cmr4gr7
Cmr3gr5
Cas10
WYL
Csx18
Cas1
Cas2
CRISPR
CRISPR: I-D
I-B
Cas2
Cas1
Cas1
Cas4
Cas6
(Cas6)
Cas3
Cas5
Cas7
Cas8b5
III-B
Cmr6gr7
Cmr5gr11
Cmr4gr7
CRISPR
CRISPR: I-D
CRISPR
Crocosphaera chwakensis CCY0110
V-K
Cas12k
III-B
Cmr4
Csx1
Cmr3gr5
Cas10
CRISPR
CRISPR
CRISPR
Crocosphaera subtropica ATCC 51142
CRISPR: V-K
V-K
Cas12k
III-B
Cas2
Cas1
Csx18
WYL
Cas10
Cmr3gr5
Csx1
Cmr4
Cmr5gr11
Cmr6gr7
CRISPR
Hybrid
(I-A,I-B)
PD_DExK
WYL
Cas6
Cas3
Cas8a1
Cas7
Cas5
Cas1
Cas2
CRISPR
CRISPR
Crocosphaera waterburyi Alani8
I-B
Cas4
Cas5
Cas7
Cas8b3
Cas3
Cas6
CRISPR
Crocosphaera watsonii (8 strains)
X No Cas genes identified
